# PKC isoforms interact with and phosphorylate DNMT1

**DOI:** 10.1186/1741-7007-9-31

**Published:** 2011-05-27

**Authors:** Geneviève Lavoie, Pierre-Olivier Estève, Nathalie Bibens Laulan, Sriharsa Pradhan, Yves St-Pierre

**Affiliations:** 1 Institut national de la recherche scientifique, INRS-Institut Armand-Frappier, Laval, QC, Canada; 2 RNA Biology Division, New England Biolabs, Ipswich, MA, USA

## Abstract

**Background:**

DNA methyltransferase 1 (DNMT1) has been shown to be phosphorylated on multiple serine and threonine residues, based on cell type and physiological conditions. Although recent studies have suggested that protein kinase C (PKC) may be involved, the individual contribution of PKC isoforms in their ability to phosphorylate DNMT1 remains unknown. The PKC family consists of at least 12 isoforms that possess distinct differences in structure, substrate requirement, expression and localization.

**Results:**

Here we show that PKCα, βI, βII, δ, γ, η, ζ and μ preferentially phosphorylate the N-terminal domain of human DNMT1. No such phosphorylation of DNMT1 was observed with PKCε. Using PKCζ as a prototype model, we also found that PKC physically interacts with and phosphorylates DNMT1. *In vitro *phosphorylation assays conducted with recombinant fragments of DNMT1 showed that PKCζ preferentially phosphorylated the N-terminal region of DNMT1. The interaction of PKCζ with DNMT1 was confirmed by GST pull-down and co-immunoprecipitation experiments. Co-localization experiments by fluorescent microscopy further showed that endogenous PKCζ and DNMT1 were present in the same molecular complex. Endogenous PKCζ activity was also detected when DNMT1 was immunoprecipitated from HEK-293 cells. Overexpression of both PKCζ and DNMT1 in HEK-293 cells, but not of either alone, reduced the methylation status of genes distributed across the genome. Moreover, *in vitro *phosphorylation of DNMT1 by PKCζ reduced its methytransferase activity.

**Conclusions:**

Our results indicate that phosphorylation of human DNMT1 by PKC is isoform-specific and provides the first evidence of cooperation between PKCζ and DNMT1 in the control of the DNA methylation patterns of the genome.

## Background

DNA methylation plays a critical role in a large variety of cellular processes by controlling gene transcription via gene silencing. Methylation in most animals occurs at the level of cytosines within the sequence CpG, although low levels of non-CpG methylation have been reported in some species. In mammals, there are two classes of DNA (cytosine-5) methyltransferases, *de novo *and maintenance methyltransferases. The *de novo *methyltransferase in mammals has two isoforms, DNMT3a and DNMT3b [1]. The maintenance methyltransferase, DNMT1, is the most prevalent DNA methyltransferase found in cells. DNMT1 has several isoforms, including an oocyte-specific isoform that lacks the first 118 amino acids [2] and a splice variant known as DNMT1b [3]. Maintenance methylation ensures the propagation of tissue-specific methylation patterns established during mammalian development. While the DNMT1 enzymes have a preference for hemimethylated DNA [4], DNMT3a and DNMT3b act on either hemimethylated or unmethylated DNA. Thus, the pattern of mammalian methylation is established and maintained by a set of at least three different DNA methyltransferases.

At present, the signaling cascade by which DNA methylation patterns are imprinted is unclear. Connections between signaling cascades and epigenetic modifications have recently been unraveled by studies showing that the phosphatidylinositol 3-kinase (PI3K)/protein kinase B (PKB) signaling pathway regulates the protein level of DNMT1, protecting it from degradation via the ubiquitin-proteasome pathway [5]. The idea that DNMT1 activity could be regulated at the post-translational level through phosphorylation by a serine/threonine kinase was supported by mass spectrometry studies, which reported phosphorylation sites on the serine and threonine residues located in the N-terminal domain [6-15]. This region of DNMT1 fulfills several regulatory functions by interacting with proteins such as LSH, EZH2, UHRF1, G9a, DMAP1 (DNMT-associated proteins), HDAC2 (a histone deacetylase), HP1β, PCNA, and Rb [16-24]. Recently, Hervouet *et al*. (2010) [25] have demonstrated that the disruption of DNMT1/PCNA/UHRF1 interactions promote a global DNA hypomethylation in human gliomas. They also found that such interactions were regulated by the phosphorylation status of DNMT1 since phosphorylation of human DNMT1 by Akt and PKC, at the specific residues serine-127/143 and serine-127 respectively, correlated with global hypomethylation [25].

The protein kinase C (PKC) family consists of ubiquitously expressed phospholipid-dependent serine/threonine kinases, which regulate a large number of physiological processes, including cell growth and differentiation. Studies on simple organisms have shown that PKC signaling paradigms are conserved through evolution from yeast to humans. This conservation underscores the importance of this family in cellular signaling and provides novel insight into PKC function in complex mammalian systems. PKC isoenzymes with differential cellular distribution, substrate specificities, and activation responsiveness are divided into three groups: the conventional PKC isoforms, which are activated by calcium, diacylglycerol, and phorbol esters (cPKCs; α, βI, βII and γ); the novel PKCs, which are activated by diacylglycerol but are calcium-insensitive (nPKCs; δ, ε, η/L (mouse/human) and θ); and the atypical PKCs, which are calcium- and diacylglycerol-insensitive (aPKCs; ζ and λ/ι (mouse/human)) [26]. Although each PKC isoform regulates a large number of downstream targets, individual members of the PKC family are, however, regulated in different ways, and an increasing number of studies indicates that they have distinct, and often opposing, roles [27-29]. In fact, it is now well accepted that each of the PKC isoforms is unique in its contribution to specific biological processes [30,31]. Whether all PKC isoforms can interact with and phosphorylate DNMT1 remains, however, unknown. Here, we have examined the ability of PKC isoforms to phosphorylate the human DNMT1.

## Results

### *In vitro *phosphorylation of human DNMT1 by PKC isoforms

**Figure 1:**
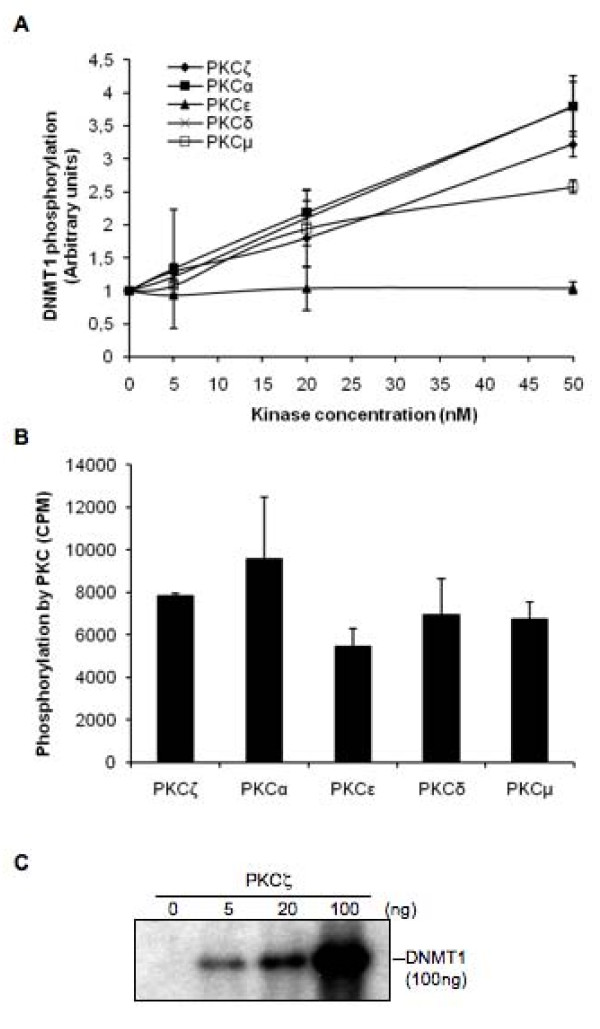
**PKC isoforms phosphorylate human recombinant DNMT1**. (**A**) Quantitative measurements of phosphorylation of 5 nM of DNMT1 in the presence of (γ-^32^P)ATP for 30 minutes at 30°C with the indicated amounts of activated recombinant human PKCα, δ, ε, μ or ζ. DNMT1 phosphorylation was quantified as the ratio of PKC activity to negative control. Data represent the average of two representative independent experiments. Bars, S.D. (**B**) PKC activity of recombinant PKC isoforms against CREB, showing that all isoforms were active. 20 nM of each PKC and 1.5 μM of CREB peptides were used for the assay and were incubated in the presence of (γ-^32^P)ATP for 30 minutes at 30°C. Bars, S.D. (**C**) Autoradiography of a SDS-PAGE showing incorporation (γ-^32^P)ATP in recombinant human DNMT1 following incubation with different amounts of human PKCζ.

Previous studies have demonstrated that human DNMT1 is phosphorylated on multiple serine and threonine amino acid residues [6-15]. Experiments using broad spectrum of inhibitors have shown that such phosphorylation on human DNMT1 is dependent on PKC activity [25]. Since PKC family members have contradictory and tissue specific roles, we have compared their ability to phosphorylate human DNMT1. Using an *in vitro *kinase assay, we found that PKCα, δ, ζ and, to a lower extent PKCμ, were all able to phosphorylate recombinant human DNMT1 in a dose-dependent manner (Figure 1A). No such phosphorylation was observed with PKCε, although this isoform showed similar activity as compared to other isoforms when tested against a CREB peptide (Figure 1B). Additional evidence of phosphorylation of recombinant full length DNMT1 by a PKC isoform was demonstrated by gel autoradiography using PKCζ as a model (Figure 1C).

**Figure 2:**
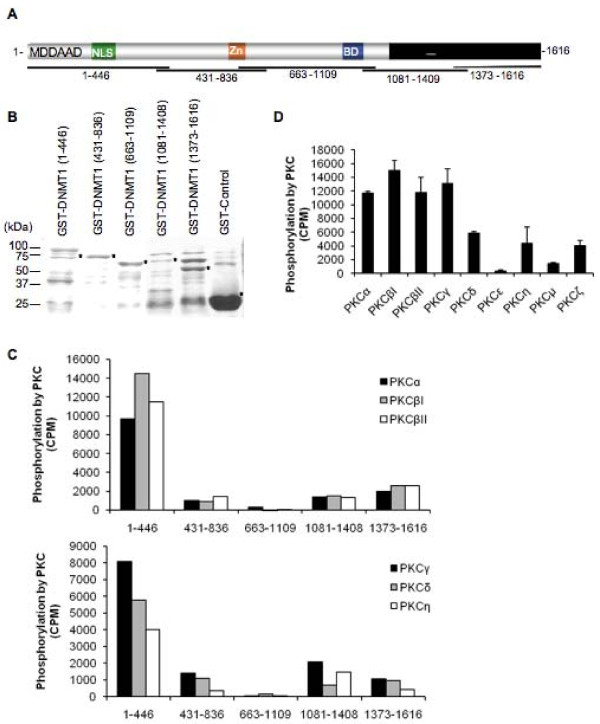
**PKC isoforms preferentially phosphorylate DNMT1 N-terminal domain**. (**A**) Diagram of DNMT1 showing the corresponding regions of the GST fusion DNMT1 fragments used for phosphorylation assays. Methylation DNA-dependent allosteric activation (MDDAAD), bromo domain (BD), and nuclear localization sequences (NLS) of DNMT1 are indicated. (**B**) Coomassie-stained gel representing GST fusion DNMT1 proteins used for phosphorylation assays. Positions of the fusion fragments are marked with an asterisk. (**C**) Phosphorylation of GST fusion DNMT1 fragments following incubation with 20 nM of activated recombinant PKCα, βI, βII, γ, δ or η using (γ-^32^P)ATP. Counts were obtained following subtraction of the negative control (GST alone). Data are representative of three independent experiments. (**D**) Phosphorylation of the GST fusion DNMT1 fragment 1 to 446 following incubation with 20 nM of activated recombinant PKCα, βI, βII, γ, δ, ε, η, μ or ζ using (γ-^32^P)ATP. Counts were obtained following subtraction of the negative control (GST alone). Data represent the average of three independent experiments that gave similar results. Bars, S.D.

**Figure 3:**
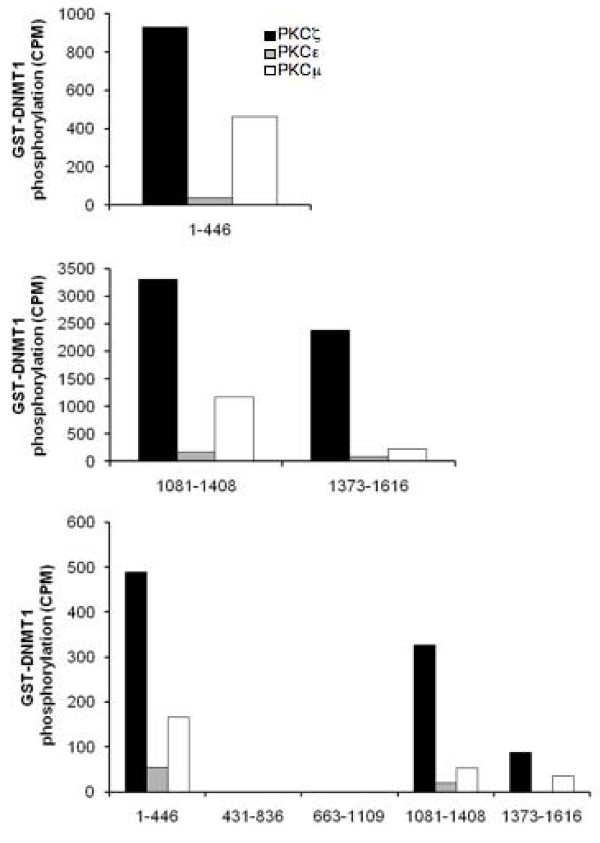
**PKCε does not phosphorylate individual domains of DNMT1**. Incorporation of (γ-^32^P)ATP by GST fusion DNMT1 fragments following incubation with 20 nM of activated recombinant PKCζ, PKCμ or PKCε. Counts were obtained following subtraction of the negative control (GST alone). Data are representative of three independent experiments.

**Figure 4:**
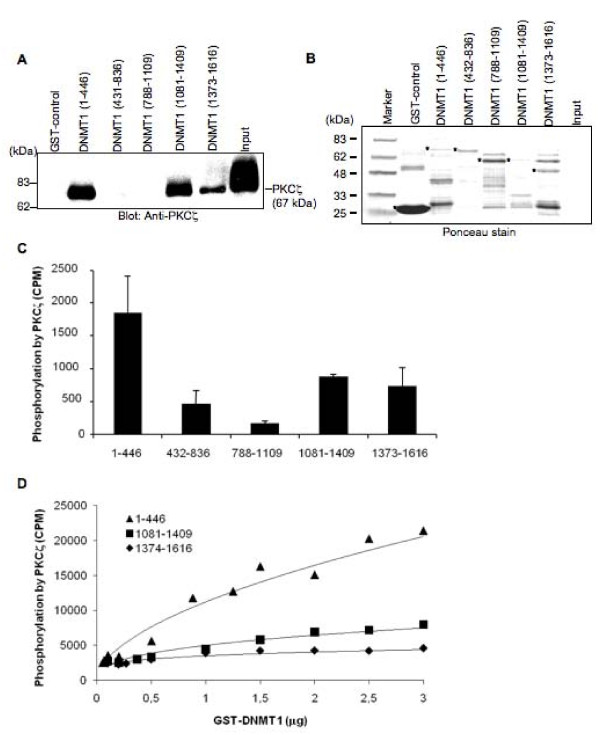
**PKCζ interacts with and phosphorylates DNMT1 fragments**. (**A**) Binding of PKCζ to GST fusion DNMT1 fragments using the pull-down procedure described in Materials and methods. Input, 10 ng of recombinant PKCζ. (**B**) Ponceau-stained transferred proteins from pull-down experiments. Positions of the fusion proteins are marked with an asterisk. (**C**) Phosphorylation of GST fusion DNMT1 fragments bound and (**D**) unbound to beads following incubation with 20 nM of activated recombinant PKCζ using (γ-^32^P)ATP. Counts were obtained following subtraction of the negative control (GST alone). Data are representative of three independent experiments. Bars, S.D.

To further compare the ability of PKC isoforms to phosphorylate DNMT1, a series of GST fusions covering the entire length of DNMT1 were challenged with recombinant PKC isoforms (Figure 2A, B). These fragments have previously been used to elucidate specific interaction between DNMT1 and accessory molecules such as hDNMT3a and hDNMT3b [32], p53 [33] and G9a [23]. Our results showed that all PKC isoforms preferentially phosphorylated the N-terminal domain (amino acids 1-446) of DNMT1 (Figure 2C, D). PKCε was inefficient in its ability to phosphorylate the N-terminal domain (Figure 2D). Such inability of PKCε to phosphorylate DNMT1 was not restricted to amino acids 1 to 446 since only negligible phosphorylation of other DNMT1 fragments was observed when compared to the ability of other isoforms (Figure 3). These results were consistent with the preferential binding of PKCζ, used here as a prototype model, with the N-terminal domain of DNMT1 (Figure 4A, B). A lower but reproducible binding was also observed between PKCζ and the C-terminal domains of DNMT1 encompassing amino acids 1081 to 1409 and 1374 to 1616 (Figure 4C, D).

### DNMT1 colocalizes with PKCζ *in vivo*

**Figure 5:**
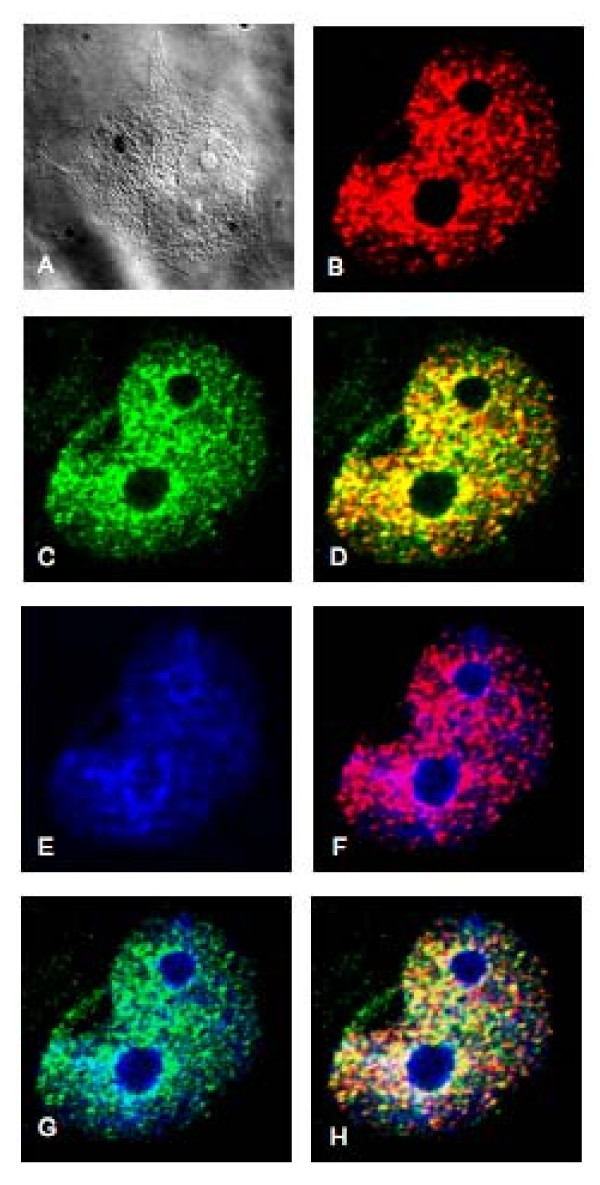
**DNMT1 and PKCζ colocalize in the nucleus of HeLa cells**. HeLa cells are shown with (**A**), DsRed.DNMT1 (red) (**B**), GFP-phosphorylated-PKCζ (green) (**C**), DsRed.DNMT1 and GFP-phosphorylated-PKCζ (merged yellow) (**D**), nucleus (blue) (**E**), merge nucleus and DsRed.DNMT1 (**F**), merge nucleus and GFP-phosphorylated-PKCζ (**G**), merge nucleus, DsRed.DNMT1, and GFP-phosphorylated-PKCζ (**H**). The construct DsRed.DNMT1 was transfected in HeLa cells 48 hours before cells fixation and permeabilization. An anti-phosphorylated-PKCζ rabbit antibody was used in combination with an anti-rabbit antibody coupled with GFP to detect endogenous activated form of PKCζ.

**Figure 6:**
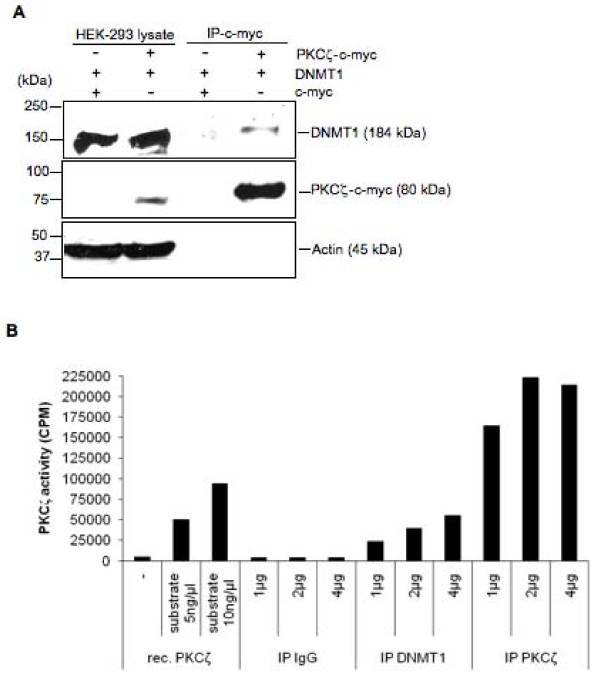
***In vivo *association between DNMT1 and PKCζ**. (A) Co-immunoprecipitation of DNMT1 and PKCζ in nuclear extracts of HEK-293 cells. The cells were transfected with DNMT1 and PKCζ-c-myc or c-myc for 48 hours and c-myc proteins were purified with immobilized anti-c-myc beads. Protein complexes were resolved by SDS/PAGE and the presence of PKCζ was demonstrated using an anti-c-myc antibody; DNMT1 and actin were revealed, respectively, using an anti-DNMT1 and an anti-actin antibody. (B) Detection of endogenous PKCζ activity in DNMT1 immunoprecipitates. Nuclear proteins from HEK-293 cells were incubated with beads prebound to an isotopic IgG antibody or antibodies against DNMT1 or PKCζ for 4 hours. After several washes, protein-bead complexes were tested for kinase activity using (γ-^32^P)ATP and PKCζ specific substrate. Data are representative of three independent experiments. rec. PKCζ, recombinant PKCζ.

Colocalization experiments were carried out by fluorescent microscopy in DsRed-DNMT1-transfected HeLa cells, which were stained with an antibody specific for the activated form of endogenous PKCζ. Red nuclear spots appeared in all of the transfectants, which was consistent with the localization of DNMT1 in the nucleus (Figure 5B, F). Green nuclear spots identifying the endogenous activated form of PKCζ were also visible in the nucleus (Figure 5C, G). Superimposition of GFP and DsRed-DNMT1 signals resulted in yellow nuclear spots, demonstrating colocalization of DNMT1 and PKCζ (Figure 5D, H). Further evidence of an *in vivo *physical interaction between DNMT1 and PKC was provided by immunoprecipitates of c-myc-PKCζ obtained from nuclear extracts and probed by Western blots with anti-DNMT1 antibody. Physical interaction between DNMT1 and PKCζ was demonstrated by the presence of DNMT1 in PKCζ-c-myc immunoprecipitates (Figure 6A). DNMT1 and PKCζ were not detected in control immunoprecipitation experiments using cells transfected with a c-myc expression control vector. Furthermore, endogenous PKCζ activity was detected in immunoprecipitates obtained using anti-DNMT1 antibody, but not from immunoprecipitates using an isotypic IgG antibody (Figure 6B). Together, these results confirmed the interaction between DNMT1 and PKCζ in HEK-293 cells.

### Overexpression of PKCζ and DNMT1 induces DNA hypomethylation of gene promoters

**Figure 7:**
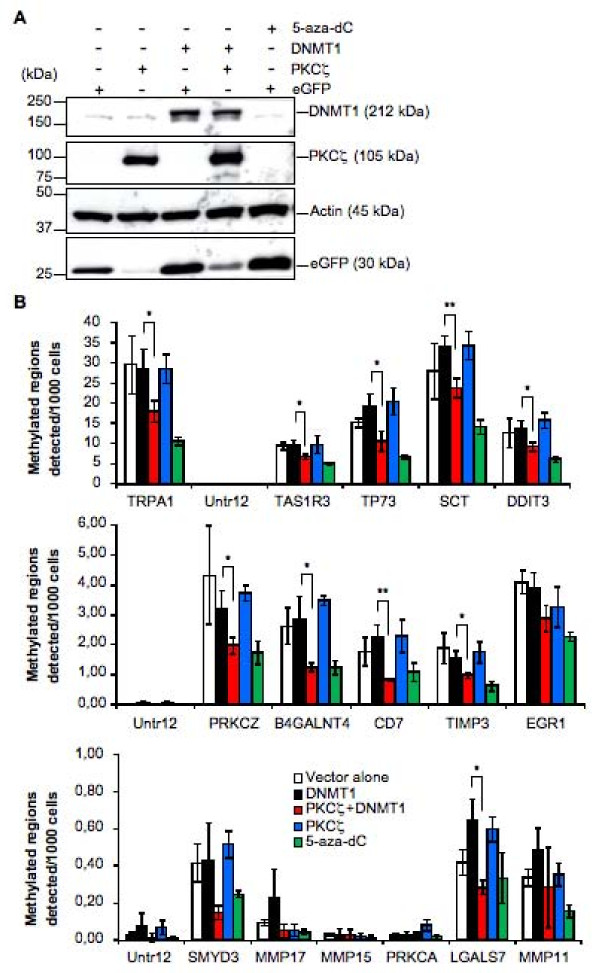
**Decrease of DNA methylation in HEK-293 cells overexpressing DNMT1 and PKCζ**. (**A**) Western blot analysis showing expression of PKCζ and DNMT1 in HEK-293 transfected cells used in the analysis of methylated DNA Ip-on-Chip described in Materials and methods. (**B**) Histograms representing the methylation status of 15 genes selected from active regions as measured by qPCR using DNA immunoprecipated with an antibody against 5-methylcytosine. Untr12 was used as a control for a negative region. TRPA1 was used as a positive control. Copy number values were normalized for primer efficiency by dividing by the values obtained using input DNA and the same primer pairs. Error bars represent standard deviations calculated from the triplicate determinations. *, *P *< 0.05; **, *P *< 0.01.

**Figure 8:**
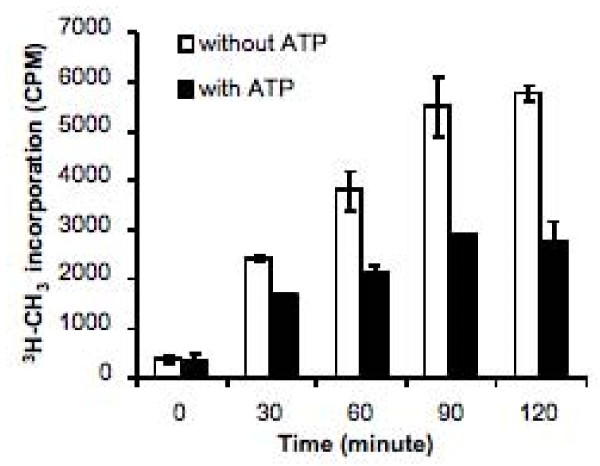
**Phosphorylation of DNMT1 by PKCζ reduces its methyltransferase activity**. Quantitative measurements of *S*-adenosyl-l-(*methyl*-^3^H)methionine integration in a DNA matrix poly(dI-dC).poly(dI-dC) by 20 nM of recombinant DNMT1 in the presence 100 ng of recombinant PKCζ incubated with or without 50 μM of ATP for different times. Data are representative of three independent experiments. Bars, S.D.

A recent study has reported that phosphorylation of DNMT1 is associated with a global DNA hypomethylation and a poor prognosis in gliomas [25]. To determine whether interactions between PKCζ and DNMT1 could also induce genome-wide changes in other cell types, the DNA methylation status on broad genomic regions were examined in HEK-293 cells overexpressing PKCζ and/or DNMT1, or control cells, including cells treated with the hypomethylating agent 5-aza-2'-deoxycytidine (5-aza-dC) (Figure 7A). For this purpose, genomic DNA was immunoprecipitated with an antibody against 5-methyl-cytosine and hybridized against Affymetrix Promoter 1.0 Tilling Arrays covering 10 to 12.5 kb regions (2.5 Kb 3' and 7.5 to 10 Kb) of 25,500 human gene promoters, with an average tilling resolution of 35 nucleotides. Analysis of the signals generated by such arrays showed an estimated 2,490 methylated regions in HEK-293 cells. Most of the methylated DNA regions identified corresponded to CpG islands (see Additional File 1). In fact, of the 2,490 methylated regions, 2,089 were in CpG islands. Fifteen regions were selected for quantitative analysis of the methylation status by quantitative PCR (qPCR) based on : 1) their distinct position on the chromosomes, 2) the presence of a CpG island within the active region, and 3) their location upstream of a gene known to be regulated by DNA methylation (although this criteria was not exclusive) (Table 1). Methylated DNA query, using specific primers for each gene showed that most, if not all, of the genes analyzed had a significant reduction in their methylation status in cells overexpressing PKCζ and DNMT1, but not in cells overexpressing either PKCζ or DNMT1 alone (Figure 7B). This reduction in the methylation status was comparable to that observed in cells treated with the hypomethylating agent 5-aza-dC. This decrease in DNA methylation status was observed on 15 genes dispersed on nine different chromosomes. Moreover, all genes, whether harboring low, medium, or high levels of methylated regions, were susceptible to the overexpression of PKCζ and DNMT1. Furthermore, *in vitro *phosphorylation of DNMT1 by PKCζ strongly reduced its methyltransferase activity (Figure 8), which was consistent with the decrease in DNA methylation observed in cells overexpressing DNMT1 and PKCζ.

**Table 1:** List of identified genes selected among active regions

Gene	**GeneBank Accession no**.	Chr #	Position of active regions	Length (bp)	# of CpG islands	Gene description
**TAS1R3**	XM_371210	1	1304459 to 1305587	1,128	1	taste receptor, type 1, member 3
**PRKCZ**	NM_002744	1	2143904 to 2144365	461	0	PKC, zeta
**TP73**	NM_005427	1	3582480 to 3584936	2,456	1	tumor protein p73
**SMYD3**	NM_022743	1	242463744 to 242466254	2,510	1	SET and MYND domain containing 3
**EGR1**	NM_001964	5	137830566 to 137831545	979	1	early growth response 1
**TRPA1**	NM_007332	8	73149159 to 73150095	936	1	transient receptor potential cation channel, subfamily A, member 1
**B4GALNT4**	NM_178537	11	352950 to 353975	1,025	0	beta1,4-N-acetylgalactosaminyltransferases IV
**SCT**	NM_021920	11	617138 to 618042	904	1	secretin
**DDIT3**	NM_004083	12	56201552 to 56202131	579	0	DNA-damage-inducible transcript 3
**MMP17**	NM_016155	12	130978656 to 130979436	780	1	matrix metalloproteinase 17
**MMP15**	NM_002428	16	56617452 to 5661737	285	1	matrix metalloproteinase 15
**PRKCA**	NM_002737	17	61729105 to 61729712	607	1	PKC, alpha
**CD7**	NM_006137	17	77870114 to 77871217	1,103	1	CD7 antigen (p41)
**LGAL7**	NM_002307	19	43973066 to 43974116	1,050	1	Galectin-7
**MMP11**	NM_005940	22	22439567 to 22439961	394	1	matrix metalloproteinase 11
**TIMP3**	NM_000362	22	31518100 to 31518635	535	0	tissue inhibitor of metalloproteinase 3

## Discussion

In the present report, we have characterized the relation between PKC isoforms and human DNMT1. More specifically, we found that: 1) PKCα, βI, βII, δ, γ, η, ζ and μ preferentially phosphorylate the N-terminal domain of human DNMT1; no such phosphorylation was observed with PKCε; 2) PKCζ and DMNT1 physically interact *in vivo *in the nucleus of HEK-293 and HeLa cells; 3) PKCζ activity could be detected in DNMT1 immunoprecipitates of endogenous DNMT1; and 4) overexpression of PKCζ and DNMT1 in HEK-293 cells induces a decrease in DNA methylation, consistent with our results showing that phosphorylation of DNMT1 by PKCζ reduces its methyltransferase activity. Overall, these results provide novel insights on the ability of PKC isoforms to play a role in controlling DNA methylation.

In a recent study, the use of broad spectrum inhibitors have suggested that phosphorylation of DNMT1 likely involves Akt and PKC [25]. Here, we provide additional evidence that PKC and DNMT1 physically interact and regulate DNA methylation. Overall, our experiments have shown that most PKC isoforms, including PKCα, β, γ, δ, η and ζ, are able to phosphorylate, albeit with different efficiency, the N-terminal region of human DNMT1. In fact, the preferential ability of PKC isoforms to interact with and phosphorylate the region encompassing amino acids 1 to 446 are consistent with previous results showing preferential phosphorylation of Serine127 [25]. Interestingly, PKCε and, to a lesser degree PKCμ, were inefficient in their ability to phosphorylate DNMT1 or its N-terminal domain. Such differential phosphorylation by PKC has often been observed. For example, phosphorylation of Ser1674 of Ca_v_1.2 α_1c_, but not Ser1928, is PKC isoform specific, as only PKCα, βI, βII, γ, δ and θ, but not PKCε, ζ and η, phosphorylate this site [34]. Although it is currently unclear why PKCε is unable to phosphorylate DNMT1, our observations provide an interesting experimental model to investigate further the functional interaction between PKC isoforms and DNMT1.

PKC participates in a multitude of cellular processes, including differentiation, proliferation, cell cycle progression and tumorigenesis [30,35]. Increasing evidence has implicated PKC isoforms in nuclear functions, suggesting that they could represent a pathway to communicate to the nucleus signals generated at the plasma membrane [36]. For example, in PC12 cells, PKCζ has been found at the inner nuclear matrix of the nucleus [37], where DNA replication gene expression and protein phosphorylation take place [38]. PKCζ has also been located in the nucleus of rat H9c2 cells during reoxygenation after ischemic hypoxia [39]. Here we provide further evidence of the presence of activated PKCζ in the nucleus of HeLa cells and of HEK-293 cells, indicating that translocation of PKCζ into the nucleus is a common mechanism not restricted to a specific cell type. Our attempts to demonstrate an interaction between endogenous DNMT1 and PKCζ by co-immunoprecipitation were, however, unsuccessful, most likely due to low expression level of DNMT1. Using a more sensitive approach, we were able to show PKCζ-specific activity in immunoprecipitates of endogenous DNMT1, supporting our hypothesis that endogenous DNMT1 and PKCζ could be found in the same complex within the nucleus. This hypothesis is also supported by our data showing that flagged DNMT1 interacts with the endogenous form of PKCζ. Whether nuclear PKCζ stands in close proximity of DNMT1, ready to act in proliferative cells, is not known. This could be, however, a very effective means to rapidly regulate DNMT1 activity when necessary. A similar paradigm has recently been proposed from studies on the regulation of DNMT1 protein stability through the coordinated interaction of an array of DNMT1-associated proteins, such as UHRF1, Tip60 (Tat-interactive protein) and HAUS (herpes virus-associated ubiquitin specific protease) [40-42].

Given its preferential ability to phosphorylate the N-terminal domain of DNMT1, PKCζ may contribute to the formation of multimolecular complexes copying the DNA methylation pattern from a parental to a replicated DNA strand. Several proteins have indeed been reported to interact with DNMT1 via its N-terminal domain, including PCNA, which recruits DNMT1 at the mammalian DNA replication forks [20,43-45]. Other proteins, such as HDAC and DMAP1 [21] initiate the formation of DNA replication complexes at the replication fork to mediate transcriptional repression. DNMT1 has also been associated with methyl-CpG-binding proteins such as MBD2, MBD3 and MeCP2 to maintain DNA methylation [46,47]. Histone methyltransferases and HP1 have been recently found to interact with DNMT1, showing a direct connection between the enzymes responsible for DNA methylation and histone methylation [23,24,48]. Furthermore, DNMT1 can interact with cell cycle regulating proteins such as Rb and p53 [22,33,49]. It is pertinent to note that PKCζ has been shown to interact with and to phosphorylate DNA-bound Sp1, thereby causing the release of the repressor p107 on the *Luteinizing Hormone Receptor *gene promoter in TSA-treated MCF-7 cells [50]. Because Sp1 interacts with HDAC1/2/mSin3A on the *Luteinizing Hormone Receptor *gene promoter in both HeLa and MCF-7 cells [51], and HDAC1/2 binds to DNMT1 [22], it is thus possible that PKCζ could interact with DNMT1 on the promoter via the Sp1/repressor complex. Additional studies will be required to test these possibilities.

Phosphorylation is one of the most common post-translational modifications occurring in animal cells. The previous observations that human DNMT1 was phosphorylated *in vivo *were indicative that at some point, DNMT1 was interacting with yet unidentified serine/threonine kinases. The results from previous mass spectrometry studies suggested that several phosphorylation sites were targeted depending on the activation status of the cell and/or the cell type [7-15], while Ser154 and Ser714 were shown to be the major phosphorylation sites in HEK-293 cells [8,12], Ser127, Ser143 and Ser714 in Jurkat cells [13] and Ser143 in lung cancer cells [15]. Although it is unclear at present whether distinct phosphorylation sites are targeted by PKC isoforms in different cell types, it is likely that Ser127 is preferentially targeted [25]. Examination of the phosphorylation profile of human DNMT1 reveals, however, the presence of several alternative phosphosites for PKC isoforms, including some located in the C-terminal regions of DNMT1. Future investigations will be necessary to identify the specific phosphorylation sites in different cell types and different states.

We found that the overexpression of PKCζ along with DNMT1 in HEK-293 cells led to a decrease in DNA methylation and that phosphorylation of DNMT1 by PKCζ reduced its methyltransferase activity *in vitro*. Our preliminary data indicate that these changes in the methylation status may not, however, be sufficient to induce or modulate gene expression. For example, no significant changes in *Egr1 *mRNA expression were observed (data not shown). This may not be surprising because DNA hypomethylation of the promoter does not always result in increased gene expression. Moreover, in cancer cells, although gene-specific hypomethylation occurs, much of the effect of global DNA hypomethylation are thought to occur through the activation of the normally dormant transposons and endogenous retroviruses present in the human genome [52]. The fact that overexpression of PKCζ alone was not sufficient to trigger genome hypomethylation may be explained, in part, by the presence of excess of PKCζ as compared to endogenous DNMT1. Unbound PKCζ might also activate signaling pathways critical for cell proliferation, differentiation and survival, such as the ERK/MAPK pathway, thereby providing a counterbalance to the negative regulation of DNMT1. It is well known that PKCζ can activate extracellular signal-regulated kinase/mitogen-activated protein kinase (ERK/MAPK) pathway in different cell types [39,53,54]. Moreover, it has been shown that inhibition of ERK/MAPK pathway lead to a decrease in DNA methylation in colon cancer cells [55].

Our data support the idea that PKC-DNMT1 interaction is important in controlling DNA methylation, possibly by regulating DNMT1 interaction with other proteins, such as UHFR1, as recently suggested [25]. This possibility is also supported by data showing that activation of PKC with phorbol ester in mouse hippocampus tissues induced a rapid demethylation of the *reelin *promoter [56]. To date, it was believed that such a role was essentially mediated through the ability of PKC to down-regulate the DNMT expression at the mRNA level [56]. Moreover, Sun *et al*., [5] have also shown that treatment of HeLa cells with a specific inhibitor of PI3K, which activates PKC, DNMT1 protein level and genomic content of methylated cytosines were decreased in a time-dependent manner without affecting the DNMT1 mRNA level. Whether phosphorylation of DNMT1 on specific residues was involved in maintaining the functional integrity of the enzyme is in fact a real possibility because mutations of one of the major phosphorylation sites of murine DNMT1, Ser515 (previously referred to as Ser514 by Glickman *et al.*, 1997) [6], has been shown to significantly reduce the *in vitro *enzymatic activity of recombinant DNMT1 [57]. Alternatively, phosphorylation of DNMT1 could affect its structural integrity, thereby reducing its DNA-binding activity, as shown by Sugiyama *et al*. via *in vitro *phosphorylation of murine DNMT1 by CK1δ [58]. It would thus be very interesting to determine, for instance, whether phosphorylation of DNMT1 modulates its ability to bind specific endogenous DNA sequences, thereby contributing to the overall genome hypomethylation. Ideally, however, such experiments will require antibodies that recognize specific PKCζ-mediated phosphorylated residues on human DNMT1. Future investigations will be needed to address this issue.

## Conclusions

This study is the first to identify PKC specific isoforms involved in the phosphorylation of DNMT1. Indeed, all PKC isoforms except PKCε, which was very inefficient, preferentially phosphorylated the N-terminal domain (amino acids 1 to 446) of DNMT1. Functional implications of DNMT1 phosphorylation by PKC isoforms have been highlighted by experiments using PKCζ as a model, which suggested possible roles in the control of DNA methylation patterns of the genome, and possibly in the control of gene expression. Based on the importance of PKC signaling in a multitude of biological processes and of a tight regulation of DNA methylation in normal cells, these findings may provide a novel strategy for cancer therapy.

## Methods

### Cell lines, reagents and constructs

The HEK-293 and the human HeLa cell lines were obtained from the American Type Culture Collection (ATCC) and maintained in Dulbecco's modified Eagle complete medium (DMEM) (supplemented with 10% (v/v) FCS, 2 mmol/L L-glutamine, 10 mmol/L HEPES buffer). All cell culture products were obtained from Life Technologies (Burlington, ON, Canada). All other reagents were purchased from Sigma Chemicals (St. Louis, MO), unless otherwise indicated. To generate pEGFP.PKCζ and pMACSK^k^.c-myc.PKCζ constructs, the PKCζ cDNA (kindly provided by Dr. Alex Toker, Department of Pathology, Harvard Medical School, Boston, MA, USA) was amplified by PCR using primers containing internal restriction sites for EcoRI and KpnI (forward primer: GAATTCATGCCCAGCAGGACCGACC; reverse primer: GGTACCCACACGGACTCCTCAGC) and XhoI and EcoRI (forward: primer: CTCGAGATGCCCAGCAGGACCGACC; reverse primer: GAATTCCACACGGACTCCTCAGC), respectively. The PCR products were then inserted in PCR4.TOPO (Invitrogen, Burlington, ON, Canada). Following enzymatic digestion with KpnI/EcoRI or XhoI/EcoRI (New England Biolabs, Ipswich, MA), the released fragment (2.12 kb) containing the coding region for PKCζ was gel-purified and ligated into pEGFP.N1 (Clontech Laboratories, Mountain View, CA) or pMACSK^k^.c-myc (C) (Miltenyi Biotec, Auburn, CA). The resulting pEGFP.PKCζ and pMACSK^k^.c-myc.PKCζ constructs were validated by sequencing and restriction enzyme analyses, as well as by Western blotting following transient transfection in HEK-293 cells. Anti-DNMT1 was obtained from New England Biolabs and the GFP antibody was obtained from Roche Applied Science (Laval, QC, Canada).

### Protein phosphorylation

GST or the fusion proteins bound to glutathione-Sepharose beads were incubated with 50 μM ATP, 1 μCi (γ-^32^P)ATP, kinase buffer (25 mM Tris-HCl (pH 7.5), 5 mM beta-glycerophosphate, 2 mM dithiothreitol (DTT), 0.1 mM Na_3_VO_4_, 10 mM MgCl_2_) and 20 nM recombinant activated PKC (α, βI, βII, δ, γ, η, μ, ζ or ε) (Cell Signaling Technology, Beverly, MA) at 37°C for 30 minutes. Beads were washed three times, resuspended in scintillation liquid and the phosphate incorporation was then measured. For phosphorylation assays using recombinant DNMT1 or fusion proteins unbound to glutathione-Sepharose, the reactions were applied on P81 phosphocellulose paper squares (Millipore, Billerica, MA) and washed three times with 0.75% phosphoric acid followed by one wash with acetone. Finally, the paper squares were put in scintillation liquid and the phosphate incorporation was measured. Otherwise, the reactions were stopped with the addition of Laemmli buffer and the samples were boiled at 98°C for five minutes. The reaction products were resolved by SDS-PAGE, and ^32^P incorporation was analyzed by autoradiography.

### GST pull-down assay and Western blot analysis

GST fusion DNMT1 and GST control proteins were expressed in *Escherichia coli *BL21 cells, as described previously [9,10]. Briefly, following induction with 0.3 mM of isopropyl-β-D-thiogalactoside (IPTG) overnight at 16°C, GST fusion proteins were purified from bacterial crude cell lysates according to the manufacturer's instructions (Pfizer-Pharmacia, New York, NY). Binding assays were performed by pre-incubating the GST or GST fusion DNMT1 proteins beads with 100 μg/ml bovine serum albumin (BSA) in a binding buffer (50 mM Tris pH 7.5, 28 μM ZnCl2, 1% Triton X-100, 220 mM NaCl, 10% glycerol) at 4°C for one hour. The beads were centrifuged, resuspended in binding buffer and incubated with 10 ng of recombinant PKCζ at 4°C for one hour. Beads were then washed three times with binding buffer containing 500 mM NaCl. The beads were mixed with 1X SDS-PAGE sample loading buffer (New England Biolabs) and incubated at 98°C for five minutes. The protein mixtures were separated on a 4 to 20% polyacrylamide gel (ISS miniplus SupraGel). The protein bands were blotted onto a nitrocellulose membrane and probed using a PKCζ antibody (Santa Cruz Biotechnology Inc., Santa Cruz, CA, USA).

### DNA methylation assay

DNA methyltransferase assays were carried out at 37°C for the indicated time in duplicate with a total volume of 25 μL of reaction mix, as described previously [4]. Briefly, 20 nM of DNMT1 (New England Biolabs) and 100 ng of PKCζ were incubated with or without 50 μM of ATP in the presence of 5 μCi of *S*-adenosyl-l-(*methyl*-^3^H)methionine (AdoMet) and 50 ng of poly(dI-dC)·poly(dI-dC) in methyltransferase buffer (50 mM Tris-HCL, pH 7.8, 1 mM Na_2_EDTA, pH 8.0, 1 mM DTT, 7 μg/ml phenylmethylsulfonyl fluoride, 5% glycerol) supplemented with 5 μg of phosphatidylserine and 5 mM MgCl_2 _to allow PKCζ activity. The reactions were stopped by transferring the tubes to an ethanol/dry ice bath, spotted on a DE81 membrane (Millipore) and processed as described previously [4].

### Immunofluorescence analysis

HeLa cells were transfected with 3 μg of DsRed-DNMT1 plasmid using Lipofectamine 2000. After 48 hours, the cells were washed with cold PBS and fixed with 4% paraformaldehyde in PBS. The cells were then permeabilized with 0.2% Triton X-100 in PBS. For endogenous phosphorylated-PKCζ labeling, the cells were first incubated overnight at 4°C with a blocking solution (BSA 5% in PBS-Tween). Antibody against phosphorylated-PKCζ (Cell Signaling Technology, Beverly, MA) was then added and incubated overnight. After several washes with PBS-Tween, the cells were incubated with an anti-rabbit secondary antibody coupled with GFP for 1 hour at room temperature and then with Hoechst 33342. Cells were dried, fixed and visualized with a Zeiss 200 M microscope (Carl Zeiss Microimaging, Thornwood, NY) with a 63x oil objective lens at 488 nm for GFP-phosphorylated-PKCζ, 568 nm for DsRed-DNMT1 fusion, and 460 nm for nuclear staining with Hoechst 33342.

### Co-immunoprecipitation

HEK-293 cells were seeded in 100 mm dish the day before transfection at a density of 2 × 10^6 ^cells/dish. Cells were transfected with pCDNA4.DNMT1 in combination with pMACSK^k^.c-myc.PKCζ or pMACSK^k^.c-myc. After 48 hours, the cells were harvested and nuclear proteins were extracted with the CelLytic NuCLEAR extraction kit. Equal amounts of nuclear lysates (500 μg) (as determined by the Bradford protein assay) and the Profound c-myc tag co-IP kit (Pierce, Rockford, IL) were used to purify c-myc-tagged PKCζ, following the manufacturer's instructions. Briefly, nuclear protein extracts were incubated with 10 μl of immobilized anti-c-myc beads with end-over-end mixing for two hours at 4°C. Complexes were washed with TBS several times and c-myc-tagged proteins were eluted with reducing sample buffer. Western blot analysis was then performed using an anti-c-myc antibody (Miltenyi Biotec) to detect c-myc.PKCζ, an anti-DNMT1 antibody to reveal DNMT1 and an anti-β-actin to detect the loading control actin.

### Western blot analysis

Cells were washed with PBS and homogenized on ice in lysis buffer (50 mM Tris pH 7.4, 150 mM NaCl, 25% glycerol, 1% Triton X-100) supplemented with a cocktail of protease inhibitors and fresh PMSF (0.5 mM) and DTT (1 mM). Equal amounts of cell lysates (as determined by Bio-Rad protein assay) were separated onto an 8% SDS-PAGE gel and transferred onto nitrocellulose membranes (Bio-Rad Laboratories, Mississauga, ON, Canada). The membranes were blocked with 5% milk in PBS/0.05% Tween-20 overnight at 4°C and then probed for two hours at room temperature with primary antibody diluted 1:5000 in PBS/0.05% Tween-20/5% milk. After several washes in PBS/0.05% Tween-20, membranes were probed with a horseradish peroxidase conjugated anti-mouse or anti-rabbit secondary antibody (Amersham Biosciences, Baie d'Urfé, QC, Canada) diluted 1:10,000 in PBS/0.05% Tween-20/5% milk for one hour at room temperature, followed by several washes in PBS/0.05% Tween-20. Detection was performed using the enhanced chemiluminescence method (Amersham Biosciences).

### Immunoprecipitation and *in vitro *kinase assay

HEK-293 cells were harvested at confluency and nuclear proteins were extracted. Antibodies against DNMT1 or PKCζ, or an isotypic IgG antibody, prebound to protein G beads (Invitrogen) were incubated with nuclear proteins in presence of protease inhibitors (at 4°C) on an orbital shaker for four hours. Proteins bound to beads were washed three times with phosphate buffer and resuspended in kinase buffer. The *in vitro *kinase assay was carried out as described earlier.

### Methylated DNA IP-on-Chip

DNA was isolated by incubating cells overnight at 50°C in SDS/proteinase K digestion buffer. Lysates were sonicated to shear the DNA to an average length of 300 to 500 bp. DNA was extracted with phenol/chloroform followed by ethanol precipitation, and then further treated with RNase and proteinase K and again ethanol-precipitated. Pellets were resuspended and the resulting DNA was quantified on a Nanodrop spectrophotometer. An aliquot of DNA (20 μg) was precleared with protein G agarose beads (Invitrogen). Methylated DNA was detected using an antibody against 5-methyl-cytosine (Abcam ab1884, San Diego, CA). After incubation at 4°C overnight, protein G agarose beads were used to isolate the immune complexes. Complexes were washed and eluted from the beads with SDS buffer. Immunoprecipitated DNA was purified by phenol/chloroform extraction and ethanol precipitation. Quantitative PCR (qPCR) reactions were carried out in triplicate on specific genomic regions using SYBR Green Supermix (Bio-Rad). The resulting signals were normalized for primer efficiency by carrying out qPCR for each primer pair using Input DNA. Immunoprecipitated and Input DNAs were amplified using either random priming or whole-genome amplification (WGA). For random priming, a fixed sequence of 17 bases containing 9 random bases at the 3' end was used in four linear amplification reactions with Sequenase (USB). Following purification, the randomly primed ChIP DNA was amplified for 30 cycles using a fixed sequence primer. For WGA, the GenomePlex WGA Kit (Sigma-Aldrich, St.Louis, MO) was used. The resulting amplified DNA was purified, quantified, and tested by qPCR at the same genomic regions as the original immunoprecipitated DNA to assess the quality of the amplification reactions. The amplified DNA was digested and labeled using the DNA Terminal Labeling Kit (Affymetrix, Fremont, CA), and then hybridized to Affymetrix GeneChip Human Promoter 1.0R arrays at 45°C overnight. Arrays were washed and scanned, and the resulting CEL files were analyzed using the Affymetrix TAS software. Thresholds were set, and the resulting BED files were analyzed using Genpathway IP (San Diego, CA, USA) analysis software, which provides comprehensive information on genomic annotation, peak metrics and sample comparisons for all peaks (intervals).

### Methylated DNA Query

Immunoprecipitated DNA was quantified at specific regions using qPCR as described above. Experimental C_t _values were converted to copy numbers detected by comparison with a DNA standard curve run on the same PCR plates. Copy number values were then normalized for primer efficiency by dividing by the values obtained using input DNA and the same primer pairs. Error bars represent standard deviations calculated from the triplicate determinations.

### Statistical analysis

Student's *t *test was used when comparing two means. The level of significance was determined at *P *< 0.05.

## Abbreviations

ATCC: American Type Culture Collection; 5-aza-dC: 5-aza-2'-deoxycytidine; CK1δ: casein kinase 1δ; CREB: cyclic AMP response element-binding protein; DNMT1: DNA methyltransferase 1; DMAP1: DNA methyltransferase 1-associated protein 1; DTT: dithiothreitol; Egr1: early growth response protein 1; ERK: extracellular signal-regulated kinase; EZH2: enhancer of zeste homolog 2; GST: glutathione S-transferase; HAUS: herpes virus-associated ubiquitin specific protease; HDAC1/2: histone deacetylase 1 and 2; HEPES: 4-(2-hydroxyethyl)-1-piperazinethanesulfonic acid; HP1: heterochromatin protein 1; IPTG: isopropyl-β-D-thiogalactoside; LSH: lymphoid-specific helicase; MAPK: mitogen-activated protein kinase; MBD: methyl-CpG-binding domain; MeCP2: methyl-CpG-binding protein 2; PCNA: proliferating cell nuclear antigen; PI3K: phosphatidylinositol 3-kinase; PKB: protein kinase B; PKC: protein kinase C; PMSF: phenylmethylsulfonyl fluoride; Rb: Retinoblastoma protein; Sp1: specificity protein 1; Tip60: tat interactive protein-60; TSA: trichostatin A; UHRF1: ubiquitin-like with PHD and ring finger domains 1; WGA: whole-genome amplification.

## Competing interests

The authors declare that they have no competing interests.

## Authors' contributions

GL, POE, SP and YSP designed the research and analysed the data. GL, POE and NBL performed the research. GL and YSP wrote the paper. All authors read and approved the final manuscript.

## Supplementary Material

Additional file 1 **ChIP-on-Chip results**. This Excel file contains the results of the ChIP-on-chip analysis. The file contains the following three sheets: the *Interval *sheet, which lists genomic segments where signals or *P*-values are above the threshold, the active regions, which lists genomic regions containing one or more Intervals, and the gene sheet, which lists all genes that have Intervals within the chosen GeneMargin. Genes can have more than one Interval within the GeneMargin. The GeneMargin is the chosen distance upstream and downstream of a gene that determines whether an Interval is associated with that gene. GeneMargins are typically set to 10,000 bp, that is, any Interval within 10,000 bp upstream or downstream of a gene is counted as being associated with that gene. Click here for file

## References

[B1] Okano M Bell DW Haber DA Li E DNA methyltransferases Dnmt3a and Dnmt3b are essential for *de novo *methylation and mammalian development Cell 1999 99 247 257 10.1016/s0092-8674(00)81656-6 10555141

[B2] Mertineit C Yoder JA Taketo T Laird DW Trasler JM Bestor TH Sex-specific exons control DNA methyltransferase in mammalian germ cells Development 1998 125 889 897 10.1242/dev.125.5.889 9449671

[B3] Bonfils C Beaulieu N Chan E Cotton-Montpetit J MacLeod AR Characterization of the human DNA methyltransferase splice variant Dnmt1b J Biol Chem 2000 275 10754 10760 10.1074/jbc.275.15.10754 10753866

[B4] Pradhan S Bacolla A Wells RD Roberts RJ Recombinant human DNA (cytosine-5) methyltransferase. I. Expression, purification, and comparison of de novo and maintenance methylation J Biol Chem 1999 274 33002 33010 10.1074/jbc.274.46.33002 10551868

[B5] Sun L Zhao H Xu Z Liu Q Liang Y Wang L Cai X Zhang L Hu L Wang G Zha X Phosphatidylinositol 3-kinase/protein kinase B pathway stabilizes DNA methyltransferase I protein and maintains DNA methylation Cell Signal 2007 19 2255 2263 10.1016/j.cellsig.2007.06.014 17716861

[B6] Glickman JF Pavlovich JG Reich NO Peptide mapping of the murine DNA methyltransferase reveals a major phosphorylation site and the start of translation J Biol Chem 1997 272 17851 17857 10.1074/jbc.272.28.17851 9211941

[B7] Beausoleil SA Jedrychowski M Schwartz D Elias JE Villen J Li J Cohn MA Cantley LC Gygi SP Large-scale characterization of HeLa cell nuclear phosphoproteins Proc Natl Acad Sci USA 2004 101 12130 12135 10.1073/pnas.0404720101 PMC514446 15302935

[B8] Molina H Horn DM Tang N Mathivanan S Pandey A Global proteomic profiling of phosphopeptides using electron transfer dissociation tandem mass spectrometry Proc Natl Acad Sci USA 2007 104 2199 2204 10.1073/pnas.0611217104 PMC1794346 17287340

[B9] Olsen JV Blagoev B Gnad F Macek B Kumar C Mortensen P Mann M Global, *in vivo*, and site-specific phosphorylation dynamics in signaling networks Cell 2006 127 635 648 10.1016/j.cell.2006.09.026 17081983

[B10] Dephoure N Zhou C Villen J Beausoleil SA Bakalarski CE Elledge SJ Gygi SP A quantitative atlas of mitotic phosphorylation Proc Natl Acad Sci USA 2008 105 10762 10767 10.1073/pnas.0805139105 PMC2504835 18669648

[B11] Cantin GT Yi W Lu B Park SK Xu T Lee JD Yates JR Combining protein-based IMAC, peptide-based IMAC, and MudPIT for efficient phosphoproteomic analysis J Proteome Res 2008 7 1346 1351 10.1021/pr0705441 18220336

[B12] Gauci S Helbig AO Slijper M Krijgsveld J Heck AJ Mohammed S Lys-N and trypsin cover complementary parts of the phosphoproteome in a refined SCX-based approach Anal Chem 2009 81 4493 4501 10.1021/ac9004309 19413330

[B13] Mayya V Lundgren DH Hwang SI Rezaul K Wu L Eng JK Rodionov V Han DK Quantitative phosphoproteomic analysis of T cell receptor signaling reveals system-wide modulation of protein-protein interactions Sci Signal 2009 2 ra46 10.1126/scisignal.2000007 19690332

[B14] Chen RQ Yang QK Lu BW Yi W Cantin G Chen YL Fearns C Yates JR Lee JD CDC25B mediates rapamycin-induced oncogenic responses in cancer cells Cancer Res 2009 69 2663 2668 10.1158/0008-5472.CAN-08-3222 PMC2697620 19276368

[B15] Tsai CF Wang YT Chen YR Lai CY Lin PY Pan KT Chen JY Khoo KH Chen YJ Immobilized metal affinity chromatography revisited: pH/acid control toward high selectivity in phosphoproteomics J Proteome Res 2008 7 4058 4069 10.1021/pr800364d 18707149

[B16] Myant K Stancheva I LSH cooperates with DNA methyltransferases to repress transcription Mol Cell Biol 2008 28 215 226 10.1128/MCB.01073-07 PMC2223296 17967891

[B17] Bostick M Kim JK Esteve PO Clark A Pradhan S Jacobsen SE UHRF1 plays a role in maintaining DNA methylation in mammalian cells Science 2007 317 1760 1764 10.1126/science.1147939 17673620

[B18] Vire E Brenner C Deplus R Blanchon L Fraga M Didelot C Morey L Van Eynde A Bernard D Vanderwinden JM Bollen M Esteller M Di Croce L de Launoit Y Fuks F The Polycomb group protein EZH2 directly controls DNA methylation Nature 2006 439 871 874 10.1038/nature04431 16357870

[B19] Sharif J Muto M Takebayashi S Suetake I Iwamatsu A Endo TA Shinga J Mizutani-Koseki Y Toyoda T Okamura K Tajima S Mitsuya K Okano M Koseki H The SRA protein Np95 mediates epigenetic inheritance by recruiting Dnmt1 to methylated DNA Nature 2007 450 908 912 10.1038/nature06397 17994007

[B20] Chuang LS Ian HI Koh TW Ng HH Xu G Li BF Human DNA-(cytosine-5) methyltransferase-PCNA complex as a target for p21WAF1 Science 1997 277 1996 2000 10.1126/science.277.5334.1996 9302295

[B21] Rountree MR Bachman KE Baylin SB DNMT1 binds HDAC2 and a new co-repressor, DMAP1, to form a complex at replication foci Nat Genet 2000 25 269 277 10.1038/77023 10888872

[B22] Robertson KD Ait-Si-Ali S Yokochi T Wade PA Jones PL Wolffe AP DNMT1 forms a complex with Rb, E2F1 and HDAC1 and represses transcription from E2F-responsive promoters Nat Genet 2000 25 338 342 10.1038/77124 10888886

[B23] Esteve PO Chin HG Smallwood A Feehery GR Gangisetty O Karpf AR Carey MF Pradhan S Direct interaction between DNMT1 and G9a coordinates DNA and histone methylation during replication Genes Dev 2006 20 3089 3103 10.1101/gad.1463706 PMC1635145 17085482

[B24] Smallwood A Esteve PO Pradhan S Carey M Functional cooperation between HP1 and DNMT1 mediates gene silencing Genes Dev 2007 21 1169 1178 10.1101/gad.1536807 PMC1865489 17470536

[B25] Hervouet E Lalier L Debien E Cheray M Geairon A Rogniaux H Loussouarn D Martin SA Vallette FM Cartron PF Disruption of Dnmt1/PCNA/UHRF1 interactions promotes tumorigenesis from human and mice glial cells PLoS ONE 5 e11333 10.1371/journal.pone.0011333 PMC2894052 20613874

[B26] Jaken S Protein kinase C isozymes and substrates Curr Opin Cell Biol 1996 8 168 173 10.1016/s0955-0674(96)80062-7 8791416

[B27] Mischak H Goodnight JA Kolch W Martiny-Baron G Schaechtle C Kazanietz MG Blumberg PM Pierce JH Mushinski JF Overexpression of protein kinase C-delta and -epsilon in NIH 3T3 cells induces opposite effects on growth, morphology, anchorage dependence, and tumorigenicity J Biol Chem 1993 268 6090 6096 8454583

[B28] Chen L Hahn H Wu G Chen CH Liron T Schechtman D Cavallaro G Banci L Guo Y Bolli R Dorn GW Mochly-Rosen D Opposing cardioprotective actions and parallel hypertrophic effects of delta PKC and epsilon PKC Proc Natl Acad Sci USA 2001 98 11114 11119 10.1073/pnas.191369098 PMC58692 11553773

[B29] Fahrmann M Kaufhold M Rieg T Seidler U Different actions of protein kinase C isoforms alpha and epsilon on gastric acid secretion Br J Pharmacol 2002 136 938 946 10.1038/sj.bjp.0704790 PMC1573419 12110618

[B30] Dempsey EC Newton AC Mochly-Rosen D Fields AP Reyland ME Insel PA Messing RO Protein kinase C isozymes and the regulation of diverse cell responses Am J Physiol Lung Cell Mol Physiol 2000 279 L429 438 10.1152/ajplung.2000.279.3.L429 10956616

[B31] Steinberg SF Structural basis of protein kinase C isoform function Physiol Rev 2008 88 1341 1378 10.1152/physrev.00034.2007 PMC2899688 18923184

[B32] Kim GD Ni J Kelesoglu N Roberts RJ Pradhan S Co-operation and communication between the human maintenance and de novo DNA (cytosine-5) methyltransferases EMBO J 2002 21 4183 4195 10.1093/emboj/cdf401 PMC126147 12145218

[B33] Esteve PO Chin HG Pradhan S Human maintenance DNA (cytosine-5)-methyltransferase and p53 modulate expression of p53-repressed promoters Proc Natl Acad Sci USA 2005 102 1000 1005 10.1073/pnas.0407729102 PMC544618 15657147

[B34] Yang L Doshi D Morrow J Katchman A Chen X Marx SO Protein kinase C isoforms differentially phosphorylate Ca(v)1.2 alpha(1c) Biochemistry 2009 48 6674 6683 10.1021/bi900322a PMC2846607 19527072

[B35] Musashi M Ota S Shiroshita N The role of protein kinase C isoforms in cell proliferation and apoptosis Int J Hematol 2000 72 12 19 10979203

[B36] Martelli AM Sang N Borgatti P Capitani S Neri LM Multiple biological responses activated by nuclear protein kinase C J Cell Biochem 1999 74 499 521 10440921

[B37] Zhou G Seibenhener ML Wooten MW Nucleolin is a protein kinase C-zeta substrate. Connection between cell surface signaling and nucleus in PC12 cells J Biol Chem 1997 272 31130 31137 10.1074/jbc.272.49.31130 9388266

[B38] Berezney R Regulating the mammalian genome: the role of nuclear architecture Adv Enzyme Regul 2002 42 39 52 10.1016/s0065-2571(01)00041-3 12123705

[B39] Mizukami Y Kobayashi S Uberall F Hellbert K Kobayashi N Yoshida K Nuclear mitogen-activated protein kinase activation by protein kinase czeta during reoxygenation after ischemic hypoxia J Biol Chem 2000 275 19921 19927 10.1074/jbc.M907901199 10777509

[B40] Achour M Fuhrmann G Alhosin M Ronde P Chataigneau T Mousli M Schini-Kerth VB Bronner C UHRF1 recruits the histone acetyltransferase Tip60 and controls its expression and activity Biochem Biophys Res Commun 2009 390 523 528 10.1016/j.bbrc.2009.09.131 19800870

[B41] Du Z Song J Wang Y Zhao Y Guda K Yang S Kao HY Xu Y Willis J Markowitz SD Sedwick D Weing RM Wang Z DNMT1 stability is regulated by proteins coordinating deubiquitination and acetylation-driven ubiquitination Sci Signal 3 ra80 10.1126/scisignal.2001462 PMC3116231 21045206

[B42] Bronner C Control of DNMT1 abundance in epigenetic inheritance by acetylation, ubiquitylation, and the histone code Sci Signal 4 pe3 10.1126/scisignal.2001764 21266713

[B43] Leonhardt H Page AW Weier HU Bestor TH A targeting sequence directs DNA methyltransferase to sites of DNA replication in mammalian nuclei Cell 1992 71 865 873 10.1016/0092-8674(92)90561-p 1423634

[B44] Liu Y Oakeley EJ Sun L Jost JP Multiple domains are involved in the targeting of the mouse DNA methyltransferase to the DNA replication foci Nucleic Acids Res 1998 26 1038 1045 10.1093/nar/26.4.1038 PMC147368 9461465

[B45] Vertino PM Sekowski JA Coll JM Applegren N Han S Hickey RJ Malkas LH DNMT1 is a component of a multiprotein DNA replication complex Cell Cycle 2002 1 416 423 10.4161/cc.1.6.270 12548018

[B46] Tatematsu KI Yamazaki T Ishikawa F MBD2-MBD3 complex binds to hemi-methylated DNA and forms a complex containing DNMT1 at the replication foci in late S phase Genes Cells 2000 5 677 688 10.1046/j.1365-2443.2000.00359.x 10947852

[B47] Kimura H Shiota K Methyl-CpG-binding protein, MeCP2, is a target molecule for maintenance DNA methyltransferase, Dnmt1 J Biol Chem 2003 278 4806 4812 10.1074/jbc.M209923200 12473678

[B48] Fuks F Hurd PJ Deplus R Kouzarides T The DNA methyltransferases associate with HP1 and the SUV39H1 histone methyltransferase Nucleic Acids Res 2003 31 2305 2312 10.1093/nar/gkg332 PMC154218 12711675

[B49] Pradhan S Kim GD The retinoblastoma gene product interacts with maintenance human DNA (cytosine-5) methyltransferase and modulates its activity Embo J 2002 21 779 788 10.1093/emboj/21.4.779 PMC125847 11847125

[B50] Zhang Y Liao M Dufau ML Phosphatidylinositol 3-kinase/protein kinase Czeta-induced phosphorylation of Sp1 and p107 repressor release have a critical role in histone deacetylase inhibitor-mediated derepression [corrected] of transcription of the luteinizing hormone receptor gene Mol Cell Biol 2006 26 6748 6761 10.1128/MCB.00560-06 PMC1592868 16943418

[B51] Liao M Zhang Y Dufau ML Protein kinase Calpha-induced derepression of the human luteinizing hormone receptor gene transcription through ERK-mediated release of HDAC1/Sin3A repressor complex from Sp1 sites Mol Endocrinol 2008 22 1449 1463 10.1210/me.2008-0035 PMC2422830 18372343

[B52] Wilson AS Power BE Molloy PL DNA hypomethylation and human diseases Biochim Biophys Acta 2007 1775 138 162 10.1016/j.bbcan.2006.08.007 17045745

[B53] Zhao Y Liu J Li L Liu L Wu L Role of Ras/PKCzeta/MEK/ERK1/2 signaling pathway in angiotensin II-induced vascular smooth muscle cell proliferation Regul Pept 2005 128 43 50 10.1016/j.regpep.2004.12.012 15721486

[B54] Yang CS Lee JS Song CH Hur GM Lee SJ Tanaka S Akira S Paik TH Jo EK Protein kinase C zeta plays an essential role for Mycobacterium tuberculosis-induced extracellular signal-regulated kinase 1/2 activation in monocytes/macrophages via Toll-like receptor 2 Cell Microbiol 2007 9 382 396 10.1111/j.1462-5822.2006.00797.x 16925784

[B55] Lu R Wang X Chen ZF Sun DF Tian XQ Fang JY Inhibition of the extracellular signal-regulated kinase/mitogen-activated protein kinase pathway decreases DNA methylation in colon cancer cells J Biol Chem 2007 282 12249 12259 10.1074/jbc.M608525200 17307743

[B56] Levenson JM Roth TL Lubin FD Miller CA Huang IC Desai P Malone LM Sweatt JD Evidence that DNA (cytosine-5) methyltransferase regulates synaptic plasticity in the hippocampus J Biol Chem 2006 281 15763 15773 10.1074/jbc.M511767200 16606618

[B57] Goyal R Rathert P Laser H Gowher H Jeltsch A Phosphorylation of serine-515 activates the Mammalian maintenance methyltransferase Dnmt1 Epigenetics 2007 2 155 160 10.4161/epi.2.3.4768 17965600

[B58] Sugiyama Y Hatano N Sueyoshi N Suetake I Tajima S Kinoshita E Kinoshita-Kikuta E Koike T Kameshita I The DNA-binding activity of mouse DNA methyltransferase 1 is regulated by phosphorylation with casein kinase 1delta/epsilon Biochem J 427 489 497 10.1042/BJ20091856 20192920

